# Long-term N fertilization reduces uptake of N from fertilizer and increases the uptake of N from soil

**DOI:** 10.1038/s41598-020-75971-0

**Published:** 2020-11-02

**Authors:** Helio Antonio Wood Joris, André Cesar Vitti, Risely Ferraz-Almeida, Rafael Otto, Heitor Cantarella

**Affiliations:** 1 ABC Foundation, Rodovia PR 151, Castro, PR 84166-981 Brazil; 2 Agribusiness Technology of the Paulista Agency – APTA, Rua São Jorge, 283 Santana, Piracicaba, SP 13411-516 Brazil; 3 Luiz de Queiroz College of Agriculture, Department of Soil Science, University of São Paulo, Av. Padua Dias, 11, Piracicaba, SP 13418-900 Brazil; 4 Agronomic Institute of Campinas, Av. Barão de Itapura, 1481 - Botafogo, Campinas, SP 13020-902 Brazil

**Keywords:** Element cycles, Environmental impact

## Abstract

Long-term supply of synthetic nitrogen (N) has the potential to affect the soil N processes. This study aimed to (i) establish N response curves to find the best balance between inputs and outputs of N over four ratoons; (ii) use ^15^N-labeled fertilizer to estimate the N recovery efficiency of fertilizer applied in the current season as affected by the N management in the previous three years. Nitrogen rates (control, 60, 120, and 180 kg ha^−1^ N) were applied annually in the same plots after the 1st, 2nd, 3rd, and 4th sugarcane cycles. Sugarcane yield, N uptake, and N balance were evaluated. In the final season, 100 kg ha^−1^ of ^15^N was also applied in the microplots to evaluate the effect of previous N fertilization on N derived from fertilizer (NDF) and N derived from soil (NDS). Sugarcane yields increased linearly with the N rates over the four sugarcane-cycles. The best balance between the input of N through fertilizer and N removal by stalks was 90 kg ha^−1^ N in both the 1st and 2nd ratoons, and 71 kg ha^−1^ N in both the 3rd and 4th ratoons. Long-term application of N reduced NDF from 41 to 30 kg ha^−1^ and increased NDS from 160 to 180 kg ha^−1^ N. A key finding is that long-term N fertilization has the potential to affect soil N processes by increasing the contribution of soil N and reducing the contribution of N from fertilizer.

## Introduction

Sugarcane is one of the most successful crops for bioenergy production^1,2^. The application of nitrogen (N) fertilizer for growing bioenergy crops is a challenging issue due to the potential for contamination of air and water by excessive fertilizer use, which can negatively impact ecosystems^3,4^. The impact of N fertilizers on atmospheric emissions of gases can offset the environmental gains achieved by the replacement of fossil fuels by bioenergy crops^5^.

One of the most important issues in the cultivation of sugarcane is the low N recovery efficiency (NRE) that barely exceeds 30% of applied N^6^. Comparatively, it is much lower than the NRE obtained for other cultivated crops, such as cereals, which present an average NRE of 50%^7,8^. The NRE is characterized as the percentage of fertilizer N recovered in plant biomass during the growing season^9^. Soluble fertilizer serves as a short-term source of N for plants^10^, while mineralization of N, which comes from soil organic matter (SOM), is a long-term source of N for arable crops^11,12^. Therefore, soil, not fertilizers, is the main source of nitrogen (N) for most cultivated crops, including sugarcane^6,10,13,14^. Strategies to maintain SOM reserves play a key role in sustaining plant production while maintaining the functionality of ecosystem services^15^.

Currently, the N recommendations for sugarcane are based on expected sugarcane yields, which may result in soil N unbalance, with applications of insufficient or excessive amounts of N^16,17^. Several management options to improve N use efficiency (NUE), defined as the amount of biomass produced relative to the amount of N applied^18–20^, in sugarcane systems have been proposed based on the complex interrelationships that exist between crop growth, N fertilizer rates, and N losses to the environment^4,21–23^. Under Australian conditions, for both sugarcane plants and ratoons, the N application rate was the most critical factor influencing NUE^4^. For Brazilian conditions, ^22^showed that reducing the N rate from 120 to 80 kg ha^–1^ of N has limited potential for lowering yields (1%) but increased the NUE (54%), a much higher value than the 14% increase achieved by modifying the N source or timing of application.

Several studies have measured the NRE by sugarcane during the same growing season^11,24^, but few studies have evaluated it in the following crop seasons. Generally, the residual effect of N from fertilizer in sugarcane is low. ^25^observed an NRE (^15^N labeled fertilizer) of approximately 30% in the first crop season, followed by 5; 4; and 4% in the second, third, and fourth years, respectively. The highest uptake in the first year and the limited recovery in the following years are indicators that immobilization plays a significant role in the dynamics of N from fertilizer in agricultural systems^12,26–28^. This is consistent with previous evidence that shows immobilization of N from fertilizer into SOM pools varying from 20 to 30% in sugarcane systems in Brazil^6,12^. This applies not only for synthetic fertilizer but also for crop residues such as straw from sugarcane harvests. Whereas the value of straw for sugarcane nutrition is limited in the short term, maintaining straw on the fields will serve as a long-term source of N and carbon for the soil^29^.

Establishing N-response field trials is required to define economic optimum N rates in sugarcane fields and helps to determine the N balance to avoid the unnecessary application of N fertilizers^6,16^. Furthermore, the use of ^15^N-labeled fertilizers allows the determination of the fate of fertilizer, and estimation of the uptake of N derived from the soil and the residual effects of N fertilization. Therefore, the use of ^15^N-labeled fertilizers is a useful approach for evaluating the effects of long-term N fertilization on the NRE in the current year since previous fertilization has the potential to affect soil N processes such as immobilization, mineralization, and soil C and N storage^15,30–32^.

We hypothesized that N fertilization aimed at achieving profitable sugarcane yields in the season of application would also have long-term effects on N nutrition. Our goal here was to (i) establish N response curves to find the best balance between N fertilizer inputs and removal by harvest over four ratoons; and (ii) to employ ^15^N-labeled fertilizer to estimate the NRE of N applied in the current season as affected by the N management in the previous three years.

## Results

### Climate and soil conditions

**Figure 1:**
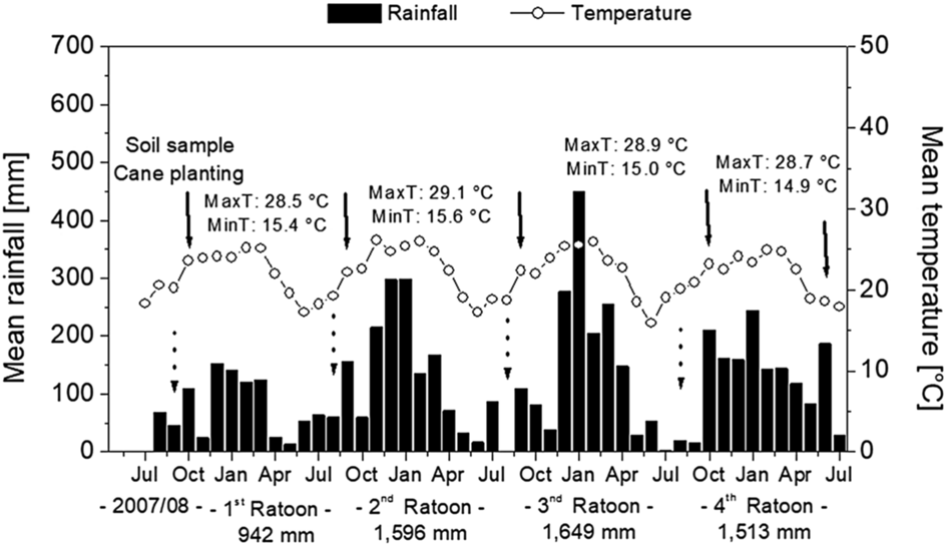
Mean precipitation (mm) and temperature (°C) during the study period. Dashed and solid arrows represent the dates of sugarcane harvests and N applications, respectively. Soil samples and cane plantings were performed in 2007/08. Annual MaxT and MinT represent maximum and minimum temperatures, respectively.

The yearly total rainfall in the 1st and 2nd ratoons were 20% lower than the mean in the 3rd (1649 mm) and 4th ratoons (1513 mm) (Fig. 1). Rainfall was concentrated in the summer (December through March), in the same period of maximum temperatures (Fig. 1).

The difference in the soil nutrient contents between the initial and final soil sampling revealed improvements in soil pH, soil organic matter, calcium, magnesium, and base saturation in the 0.0–0.2 m soil depth (Table 1S). However, the contents of phosphorous, potassium, exchangeable acidity (H + Al), and cation exchange capacity reduced over the four ratoons (Table 1S).

### Sugarcane yield

**Figure 2:**
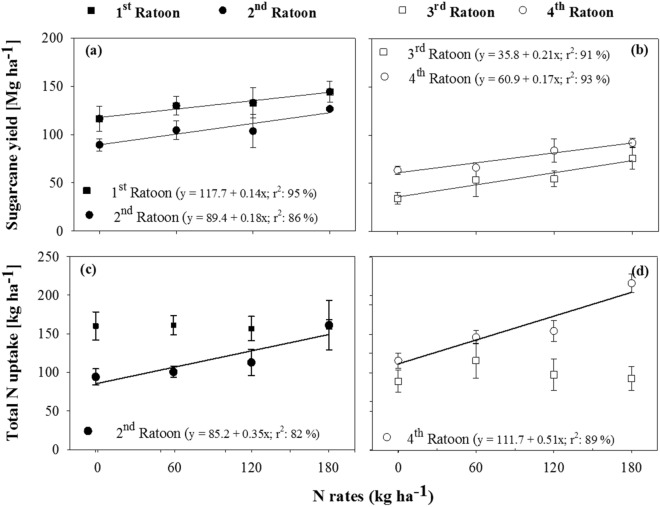
Sugarcane yields (**a**, **b**; Mg ha^−1^) and total N uptake (**c**, **d**; kg ha^−1^) in response to annual N rates (control; 60; 120; and 180 kg ha^−1^ N) applied in four consecutive ratoons (1st; 2nd; 3rd; and 4th ratoon). Regression equations (*P* ≤ 0.05) were fit to the response to N rates. Bars indicate mean standard deviation.

The N rates fitted a positive linear response against sugarcane yields in all crop seasons (R^2^ ≥ 80%; *P* ≤ 0.05), with average yields of 131; 106; 55; and 76 Mg ha^−1^ in the 1st, 2nd, 3rd, and, 4th ratoons, respectively (Fig. 2a,b).

Total N uptake increased linearly by N rates in the 2nd and 4th ratoons (R^2^ ≥ 80%; *P* ≤ 0.1) (Fig. 2c,d; Table 2S). There was also a linear correlation between N uptake and sugarcane production with r values of 0.76 and 0.82, respectively in the 2nd and 4th ratoons (*P* ≤ 0.05). In contrast, N rates exhibited no effect on N uptake in the 1st and 3rd ratoons with a mean of 160.1 and 98.9 kg ha^−1^, respectively. Moreover, there was no significant correlation between N uptake with sugarcane production in those seasons (*P* ≤ 0.05; Fig. 2c,d; Table 2S).

**Figure 3:**
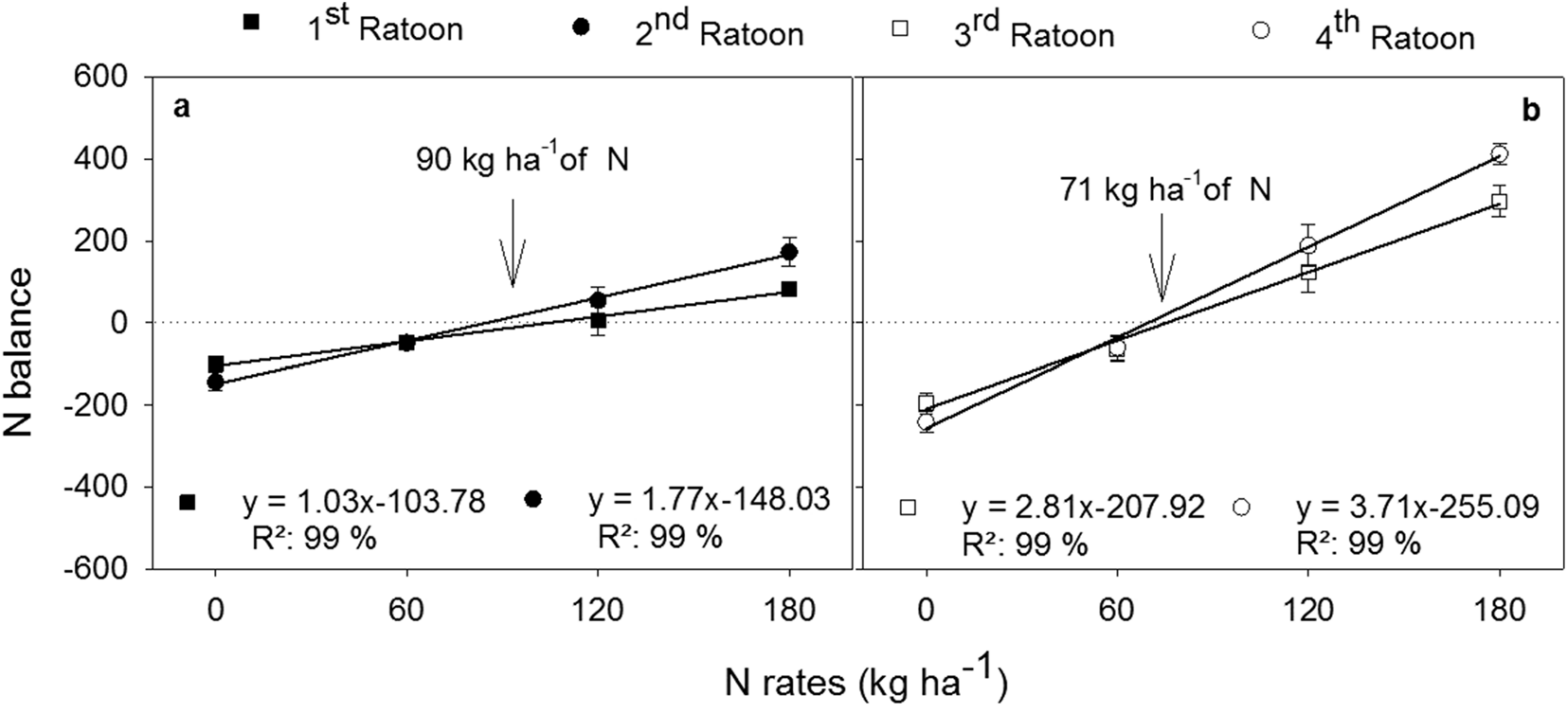
Nitrogen balance between N inputs by annual rates of N fertilizer (control; 60; 120; and 180 kg ha^−1^ of N) and N export by sugarcane harvest in four consecutive ratoons (**a**: 1st and 2nd; **b**: 3rd and 4th ratoons). The N balances for the 2nd to 4th ratoons refer to cumulative inputs and exports over the years. Optimal rates were obtained by derivation of the linear equations.

The balance between N inputs through fertilizer and N removal by stalks revealed the need to apply 90 kg ha^−1^ of N in the 1st and 2nd ratoons, and to apply 70 kg ha^−1^ of N in the 3rd, and 4th ratoons to maintain a neutral balance (Fig. 3). Although sugarcane yields improved linearly by N rates, the results of N balance indicate that the application of N rates above that threshold could cause over-fertilization with N, increasing the potential for N losses in the long-term.

### Nitrogen recovery efficiency

In the final crop cycle, the isotopic balance method revealed a reduction in NDF in the plots that received previous N fertilization (Table 1). In the unfertilized plots, NDF totaled 41 kg ha^−1^, while in the plots that received long-term N fertilization (varying from 60 to 180 kg ha^−1^ N per year), the NDF was reduced to 31–32 kg ha^−1^, with a mean difference of 9 kg ha^−1^ (Table 1). In contrast, NDS was higher in the fertilized plots (181 kg ha^−1^ averaged among N rates) and lower in the unfertilized plots (161 kg ha^−1^), represented by an increase of 20 kg ha^−1^ of N derived from soil (Table 1).

**Table 1:** Nitrogen derived from fertilizer (NDF), N derived from soil (NDS), and total N uptake by sugarcane affected by N applications in the previous 3 years.

Cumulative N applied in the previous 3 years	NDF	NDS	Total
kg ha^−1^	kg ha^−1^		
0	41a	161a	202b
180	32b	181b	213a
360	31b	182b	213a
540	32b	182b	213a
P-value	≤ 0.05	≤ 0.05	≤ 0.05

NDF was calculated by the isotopic balance method with an N rate of 100 kg ha^−1^ applied in the 4th year to microplots. In columns, different lowercase letters indicate differences by the LSD-Test (*P* ≤ 0.05). Number of replications = 4.

The aggregated values resulted in an increase in total N uptake from 202 kg ha^−1^ N in the unfertilized plots to 213 kg ha^−1^ in the fertilized plots (P ≤ 0.05; Table 1). Most of the fertilizer N from soil and fertilizer were accumulated in the tops, followed by accumulations in the stalks and dry leaves with a respective mean of 121.4; 70.1; and 19.0 kg ha^−1^ (Table S3).

## Discussion

The positive effects of N fertilization on ratoon yields were expected and have been described previously in studies in the same region^33–36^. Nonetheless, as commonly observed, the response of ratoons to N fertilization was still erratic under Brazilian field conditions^33^. A linear response of the first ratoon to N rates of up to 175 kg ha^−1^ was demonstrated by^34^, while^12^ found responses up to 150 kg ha^−1^ in a clay soil. However, more often, the responses of sugarcane to N fit a quadratic model^6,37,38^. Recent studies of sugarcane in Brazil report a limited response of sugarcane to increases in N rates^39^. In most cases, unresponsive sites have a history of previous applications of organic byproducts of the sugarcane industry, such as filter cake and vinasse, and/or rotation with legumes before the establishment of sugarcane^6^. This was not the case in our study since sugarcane exhibited a linear response to N in all years, indicating that the maximum yield was not achieved.

The positive response to N can also be related to adequate rainfall during all seasons. The least accumulated rainfall occurred in the 1st ratoon; nevertheless, the precipitation was still sufficient to provide high yields and a positive response to N. The high clay content of the soil, enhancing soil water storage, and the adaptability of the variety IAC 92–1099 for water shortage^40–42^, could have limited the negative effect of water stress on sugarcane growth. The decline in yield observed overtime was not correlated to accumulated rainfall, which was higher in the lowest yielding cropping cycles. The decline in yield was probably associated with a decline in sugarcane vigor with aging^44,45^, as well as soil compaction during mechanical harvesting that injures roots and increases failure during sugarcane sprouting. The decline in soil P and K contents over the four years (Table 1S) may have also contributed to the decline in yield because both nutrients are required in large amounts during sugarcane fertilization^43^.

The expected yield concept is commonly employed to recommend N applications for sugarcane in Brazil^6^. In the state of São Paulo, the usual recommendation is to apply 1.1 kg N per Mg^−1^ of stalk yield expected (calculated from^43^). Considering the mean yield obtained in the seasons and a ratio of 1.1 kg N per Mg^−1^, this would result in N recommendations of 143, 117, 60, and 84 kg ha^−1^ of N for the 1st, 2nd, 3rd, and 4th ratoons, respectively. The recommended N levels for the first two crop seasons are somewhat higher than those obtained by the N balance method, e.g., 90 kg ha^−1^ for the 1st and 2nd ratoons, but are similar to the 71 kg ha^−1^ recommended for the 3rd and 4th ratoons, respectively. The application of the optimal rate can reduce the amounts of N not taken up by plants and avoid the soil N losses by nitrate leaching^44^ and nitrous oxide emissions^45^.

Recommending fixed N rates or rates based on expected yield can result in excess N in the system since it is difficult to forecast the yield (especially for rainfed systems), and usually, the attained yield is lower than the expected yield. In the recommendation method proposed by^16^, an N-replacement strategy was suggested to improve profitability and reduce the environmental impacts associated with excessive N inputs. For that approach, only the N removed from the field, plus extra N to cover losses, would be recommended, as opposed to a fixed amount of N based on the expected yield. The results presented herein corroborate^16^ in demonstrating the economic and environmental advantages of progressing from the expected yield approach to a replacement concept.

The NRE fits within the range observed in other sugarcane studies^6,24^, confirming the low uptake of N from fertilizer for sugarcane cropping systems. In our study, we did not measure the N fractions in the root systems; therefore, we may have slightly underestimated NRE. Roots represent approximately 15 to 19% of the plant biomass at the active growing stages in sugarcane, but only 4 to 11% close to plant maturity^46,47^. Otto et al.^48^ found that sugarcane roots accumulated approximately 12 kg N ha^−1^ at harvest, as an average of several treatments, in sugarcane grown in areas close to that of the present study.

The improvement of NDF in the nonfertilized plots (40.7 kg ha^−1^, Table 1) can probably be associated with depletion of inorganic N content in soil promoted by the absence of N fertilization in the previous years. This may have stimulated the uptake of fertilizer N by plants rather than the N immobilization into SOM pools in the depleted soil conditions of the control treatment. The lower NDS in the control plots reveals a depletion of soil fertility over time, which reduces the power of the soil for supplying N by the long-term uptake of N from the growing plants. To supply adequate N to crops, soil fertility needs to be maintained at an appropriate level^49,50^. On the other hand, the reduction of NDF in fertilized plots (mean of 31 kg ha^−1^ across all N rates) can be explained by an increase of N immobilization into organic forms, following more intense microbial activity and N cycling promoted by an adequate supply of N in previous years.

The increase in net mineralization by long-term synthetic N fertilization is well documented in the literature^31,32^. The enrichment of soil N pools has the potential to modify the microbial process of C and N transformation in soils^51^, which may ultimately affect NRE. Several interactions between soil N processes can be affected by a continuous supply of inorganic N. Long-term additions of inorganic N can affect soil organisms as well as C and N cycling, directly altering soil N availability and soil pH or modifying plant interactions with the soil^51–55^. The input of N in the soil also tends to stimulate soil organic matter decomposition, which is called soil priming effect^28,56,57^.

The soil and other sources provided approximately 80 to 85% of the total N uptake by sugarcane, whereas the remaining (~ 15–20%) came from fertilizer N (calculated from Table 2S). Such values are in agreement with previous studies of sugarcane, proving that soil is the main source of N in sugarcane fields^6,10,13,14^. Since NDS is the main source of sugarcane nutrition and long-term N fertilization improved NDS, such results demonstrate the need for adequate N management of sugarcane fields to maintain the potential of soil to supply N. This reinforces the importance of maintaining crop residues over the soil surface in green sugarcane areas. Although these residues do not provide a ready source of N to sugarcane crops in the short term, they can contribute to SOM reserves and N nutrition of the crop in the long-term^12,29,58^.

Despite the evidence shown here that previous N fertilization increases the contribution of soil N for the next crop, assessing the potential of SOM to supply N to sugarcane remains a challenge. Recently^39^, tested 15 indices of soil N availability in several N-rate trials conducted from 2006 to 2013 in the same region as the present study and concluded that none of the methods could reliably predict sugarcane response to N fertilization. Apparently, until now, the replacement method for recommending fertilizer N management is the most promising alternative to ascertain sufficient N rates for sugarcane production.

## Conclusions

The N derived from soil was responsible for 80–85% of the total uptake of N in sugarcane, while 15–20% was derived from fertilizer. Long-term application of N fertilization affected soil N processes. The lack of N fertilization in previous years caused sugarcane to take up more N from fertilizer; however, long-term N fertilization increased the uptake of N from the soil. This was observable due to the use of ^15^N-labeled fertilizer that helped to explain the highly variable responses to N fertilization for this crop. Long-term N fertilization seems to enhance soil microbial activity and potentialize the priming effect. The adequate management of N fertilization in sugarcane is required to avoid unnecessary N usage; however, establishing guidelines for N recommendations remains a challenge.

## Material and methods

### Site characterization

A trial was established in a sugarcane field located in Piracicaba, Brazil (22° 43′ S; 47° 38′ W; 546 m), from 2009 to 2012. The climate is classified as Aw (Tropical savanna climate with dry-winter characteristics) by the Köppen classification (rainy, warm summer and dry, cold winter). The soil was classified as a Typic Hapludox^59^, corresponding to a Latossolo Vermelho distroférrico in the Brazilian Classification System^60^; with a clay texture and a particle size distribution of 568, 136, and 295 g kg^−1^ of clay, silt, and sand, respectively (0.0–0.4 m depth).

The area had been cultivated with sugarcane for the previous 20 years. Sugarcane (IAC 92-1099 variety) was planted in March 2007, using 15–20 viable buds per meter of row. The IAC 92-1099 variety is well-adapted to tropical conditions with high-yield potential^42^. The soil was prepared using a conventional system (plowing, disking, harrowing, and furrowing) with the incorporation of dolomitic lime before planting (3.5 Mg ha^−1^), aiming to achieve 70% of base saturation. Fertilizations were performed in the furrows with an application of 48, 168, and 96 kg ha^−1^ of N, P_2_O_5_, and K_2_O, respectively, with ammonium phosphate as a source of N and P, and potassium chloride as a source of K. Application of liming and fertilizers followed the recommendations for the region^43^. In May 2008, the plant cane was harvested mechanically without burning, producing 216 Mg ha^−1^ of stalks, which is a great yield compared to the regional average of 78 Mg ha^−1^ of stalks^61^. In September 2009, after the harvest of the 1st ratoon and before the N treatments were established, dolomitic lime (4 Mg ha^−1^) and gypsum (2 Mg ha^−1^) were applied to neutralize the soil acidity, and supply S. The nutrient contents in soil were continuous monitored, with application of fertilizers when necessary, to maintain the adequate conditions during the sugarcane cropping cycle.

### Experimental design

The experimental design was a randomized block with four replications, consisting of three rates of N (60, 120, and 180 kg ha^−1^ of N) plus an additional control, which were applied in the 1st, 2nd, 3rd, and 4th ratoons, respectively (Fig. 1S). Each experimental unit consisted of ten 30-m long sugarcane double rows, spaced at 0.9 m between the narrow lines and 1.4 m between the wide lines. The same rates of N were applied successively over the four ratoons, from 2008 through 2011 (Fig. 1S). In October 2011, after the harvest of the 3rd ratoon, the plots were split. In the 4th ratoon, half of the plots remained with the original N rate treatment, and half (5 rows of sugarcane 15-m long) received a fixed rate of 100 kg ha^−1^ of N, including the unfertilized control of the previous years. Inside each of the later plots, microplots were established where ^15^N-labeled fertilizer was applied at a rate of 100 kg ha^−1^ N (5.29% of ^15^N atoms). Each microplot unit was comprised of three sugarcane rows 2-m long and 1.4-m apart; ^15^N labeled ammonium sulfate was applied in a 0.2-m wide band close to the central row, while the neighboring rows received unlabeled fertilizer. Therefore, in the 4th ratoon, we had plots to measure the cumulative effect of the N application (original fertilization plots) and microplots where the residual effect of previous fertilization could be evaluated using ^15^N as a tracer.

Ammonium nitrate was used in the first three ratoons and ammonium sulfate in the 4th ratoon, because ^15^N-labeled ammonium nitrate was not available for the microplots. Thus, ammonium sulfate was applied to all plots. The N applications were performed after the harvest (without burning) of the sugarcane stalks of the previous year and after the first rainfall at the beginning of each cycle. The fertilizer treatments were surface applied in bands approximately 0.2 m from the row, over the straw blanket from the previous harvest. Other nutrients were applied every year to all plots, supplying 150 kg ha^−1^ of K_2_O (potassium chloride) and 50 kg ha^−1^ of P_2_O_5_ (triple superphosphate). All fertilizations followed the sugarcane recommendations for São Paulo state^43^.

### Sugarcane yield measurements

Sugarcane harvests were performed manually over the whole experimental area at 338, 350, 344, and 398 days of plant development in the 1st, 2nd, 3rd, and 4th ratoons, respectively. Plants were separated into stalks, leaves, and tops. The stalks were weighed to estimate sugarcane yield (Mg ha^−1^ of stalks). Samples from a 2-m row of tops, stalks, and leaves were weighed and dried (65 °C) to determine the dry matter biomass of each plant component. Subsamples were ground in a Willey type mill, and the N content was determined^62^. The N content of each component was calculated using the dry biomass and N concentrations. The N balance was calculated for each treatment and crop cycle, according to Eq. (1), based upon N applied by fertilizers and the N removed by stalks with the harvesting.

1 $$\mathrm{N balance }\left(\mathrm{kg }{\mathrm{ha}}^{-1}\right)= {\mathrm{N}}_{\mathrm{applied}} {-\mathrm{ N}}_{\mathrm{Removed}} \, {\mathrm{by}} \, {\mathrm{stalks}}$$

### N recovery efficiency

The N recovery efficiency was evaluated in the 4th ratoon with data of the ^15^N fertilizer microplots to determine the N derived from fertilizer (NDF) and N derived from soil (NDS) in the last crop season.

At the maturation of the 4th ratoon, plants from the ^15^N-microplot were collected in 1 m of the row at the center of the microplot as well as in 1 m of the adjacent rows at both sides. Plants from the adjacent rows were collected to detect the ^15^N taken up by neighboring plants, as suggested by^63^. Plant samples were separated (tops, stalks, and leaves), weighed, and dried to measure dry matter (65 °C). The N contents and N accumulation were determined according to^62^. The ^15^N abundance was quantified using a mass spectrometer coupled to an automatic IRMS analyzer (ANCA GSL model, Sercon Co., Crewe, UK), according to the recommendations of^63^ using a direct approach.

The dry biomass of each plant component, the N content, the abundance of ^15^N, the N rates, and the initial abundance of ^15^N in the fertilizer were used to estimate NDF, on a kg ha^−1^ basis. The difference between the total uptake of N per plant component and the NDF was assumed to be the NDS. We calculated NDF and NDS for each sugarcane row (central-row and adjacent rows), and the amounts of NDF and NDS obtained in the adjacent rows were added to the amount obtained in the central row. We calculated the NDF and NDS for each plant component (dry leaves, tops, and stalks), and the results are presented in detail in Tables 2S and 3S (Supplementary Material). In the main document (Table 1), the values were aggregated to present only the NDF and NDS levels for the whole plant. Calculation procedures were similar to those performed by^13,63^. The NDF was calculated using the following equations. Where, *a* is the abundance of ^15^N atoms in the sample; *b* is the abundance of ^15^N atoms in the fertilizer; *c* is the natural abundance of ^15^N atoms (0.366%); AR and CR refer to the N in the adjacent rows and in the central row, respectively; Total N is the total N of the above-ground biomass in kg ha^−1^.

2 $$NDF (\%)= \left[\frac{\mathrm{a } - \mathrm{ c}}{\mathrm{c } - \mathrm{ b}}\right] 100$$

3 $${\mathrm{NDF}}_{\mathrm{CR}} ({\mathrm{kg ha}}^{-1}\mathrm{ of\,\, N })= \left[\frac{{\mathrm{NDF}}_{\mathrm{\%}}}{100}\right]\mathrm{total \,\,N}$$

4 $${\mathrm{NDF}}_{\mathrm{AR}} ({\mathrm{kg ha}}^{-1}\mathrm{ of\,\, N}) = 2 \left[\frac{{\mathrm{NDF}}_{\mathrm{CR}}}{100}\right]\mathrm{ total\,\, N}$$

5 $$\mathrm{Total NDF }({\mathrm{kg ha}}^{-1}\mathrm{ of \,\,N}) ={\mathrm{NDF}}_{\mathrm{CR}} + {\mathrm{NDF}}_{\mathrm{AR}}$$

Soil samples were collected from six positions (replicates), before sugarcane planting in February 2007 (initial soil sampling), and after the 4th ratoon (final soil sampling), at the soil depths of 0–0.2 and 0.2–0.4 m. In the 4th ratoon, soil samples also were collected from the sugarcane rows in the control plots (to avoid interferences of fertilizer N). Samples were submitted to chemical analysis according to^62,64^. The results were used to calculate the difference in the contents of nutrients between initial and final sampling.

### Statistical analysis

The assumptions of normality and homogeneity of variance were evaluated by the Shapiro Wilk-Test and the Bartlett-Test, respectively. Data were submitted to an analysis of variance (ANOVA) based on the F-test. When the F-Test was significant (P ≤ 0.05), the effects of N rates and balance were evaluated by the Regression test using a linear model (P ≤ 0.05), while NDF, NDS, and total N uptake by sugarcane were compared by the LSD-test (P ≤ 0.05). Pearson’s correlation was further used to explain the relationships between variables (P ≤ 0.05). Statistical analysis was performed using R Statistical Software (version 4.0.0; R Foundation for Statistical Computing).

## Supplementary information

Supplementary Information.

## Data Availability

All data generated or analyzed during this study are included in this published article.
