# Supplementary material for: Long-term N fertilization reduces uptake of N from fertilizer and increases the uptake of N from soil
Source: Sci Rep. 2020 Nov 2;10:18834. doi: 10.1038/s41598-020-75971-0 (PMC7606474; doi:10.1038/s41598-020-75971-0)
Supplement: Supplementary file 1 — Supplementary Information. [file 41598_2020_75971_MOESM1_ESM.docx]

**Supplementary Material**

Scientific Reports

Headings: Nitrogen dynamics in soil

**Long-term N fertilization reduces uptake of N from fertilizer and increases uptake of N from soil**

Helio Antonio Wood Joris^1^, André Cesar Vitti^2^, Risely Ferraz-Almeida^3^, Rafael Otto^3 *^, Heitor Cantarella^4^

^1-^ ABC Foundation, Rodovia PR 151, 84166-981, Castro - PR, Brazil. E-mail: hwjoris@gmail.com (ORCID: 0000-0001-8637-5273).

^2 -^ Agribusiness Technology of the Paulista Agency – APTA, Rua São Jorge, 283 Santana, 13411-516, Piracicaba - SP, Brazil. E-mail: acvitti@apta.sp.gov.br (ORCID: 0000-0003-4240-3353).

^3-^ University of São Paulo, Luiz de Queiroz College of Agriculture, Department of Soil Science. Av. Padua Dias, 11, 13418-900, Piracicaba – SP, Brazil. E-mail: [rizely@gmail.com](mailto:rizely@gmail.com) (ORCID: 0000-0003-0577-3961); [rotto@usp.br](mailto:rotto@usp.br) (ORCID:0000-0003-1472-298X).

^4-^ Agronomic Institute of Campinas, Av. Barão de Itapura, 1481 - Botafogo, 13020-902, Campinas - SP, Brazil. E-mail: [cantarella@iac.sp.gov.br](mailto:cantarella@iac.sp.gov.br) (ORCID: 0000-0002-1894-3029).

*Corresponding author: Rafael Otto (rotto@usp.br; +55 19 3417-2104)

**Tab. 1S** Initial (baseline) and final soil chemical analysis.

| **Soil layer** | **pH** | **SOM** | **P** | | **Ca** | | **Mg** | | | **K** | **H+Al** | | **CEC** | | | **BS** | | **B** | | **Cu** | | **Fe** | | **Zn** |
| --- | --- | --- | --- | --- | --- | --- | --- | --- | --- | --- | --- | --- | --- | --- | --- | --- | --- | --- | --- | --- | --- | --- | --- | --- |
| m |  | g dm^-3^ | mg dm^-3^ | | ^________________^ mmol_c_ dm^-3 _________________^ | | | | | | | | | | | % | | ^___________^ mg dm^-3 _______________^ | | | | | | |
| Initial soil sampling | | | | | | | | | | | | | | | | | | | | | | | | |
| 0.0–0.2 | 4.0 | 22.1 | | 8.0 | | 10.0 | | 5.0 | 2.4 | | | 72.0 | | 89.4 | 20.0 | | 0.29 | | 1.0 | | 37.0 | | 0.6 | |
| 0.2–0.4 | 4.0 | 19.0 | | 5.1 | | 4.1 | | 4.1 | 1.7 | | | 68.2 | | 78.1 | 14.0 | | 0.23 | | 0.8 | | 31.1 | | 0.4 | |
| Final soil sampling | | | | | | | | | | | | | | | | | | | | | | | | |
| 0.0–0.2 | 4.9 | 27.5 | | 5.3 | | 18.5 | | 11.6 | 1.7 | | | 51.0 | | 82.8 | 31.9 | | 0.2 | | 3.0 | | 46.8 | | 1.4 | |
| 0.2–0.4 | 4.6 | 19.4 | | 2.1 | | 9.9 | | 4.8 | 0.8 | | | 60.4 | | 78.0 | 17.6 | | 0.2 | | 3.0 | | 29.3 | | 1.0 | |
| Balance | | | | | | | | | | | | | | | | | | | | | | | | |
| 0.0–0.2 | +0.9 (+19%) | +5.5  (+20%) | | -2.7  (-33%) | | +8.5  (+46%) | | +6.6  (+57%) | -0.7  (-28%) | | | -21.0  (-29%) | | -6.2  (-7%) | +11.9  (+37%) | | 0.0  (0%) | | +2.0  (+67%) | | +9.8  (+21%) | | +0.8  (+57%) | |
| 0.2–0.4 | +0.6  (+13%) | +0.4  (+2%) | | -3.0  (-60%) | | +5.9  (+59%) | | +0.8  (+17%) | -0.9  (-50%) | | | -7.6  (-11%) | | 0.0  (0%) | +3.6  (+20%) | | 0.0  (0%) | | +2.2  (+73%) | | -1.8  (-6%) | | +0.6  (+61%) | |

Initial analysis was collected before sugarcane planting, and the final analysis was collected after the 4^th^ ratoon. pH in CaCl_2_ (0.01 mol L^-1^); soil organic matter (SOM); available phosphorus (P); exchangeable calcium (Ca), magnesium (Mg) and potassium (K); total acidity (H+Al); cation exchange capacity (CEC); base saturation (BS); boron (B), copper (Cu), iron (Fe), and zinc (Zn). The soil N was not monitored due to natural variations in N dynamics and because N is not included in the routine soil analysis in Brazil. The balance is the difference between the content of nutrients in each soil layer in the initial and final soil analysis; values between brackets represent the difference in percentage.

**Tab. 2S** Sugarcane stalk yield and N accumulation in plant compartments as related to N rates applied over four consecutive ratoon cycles^1^.

| **N rates (kg ha^-1^)** | | | | | |  | **Crop cycle (ratoon)** | | | | | | |
| --- | --- | --- | --- | --- | --- | --- | --- | --- | --- | --- | --- | --- | --- |
| **Annual** | | |  | **Accumulated** | |  | **1^st^** |  | **2^nd^** |  | **3^rd^** |  | **4^th^** |
| **Stalk yield (Mg ha^-1^)** | | | | | | | | | | | | | |
| 0 |  |  | | 0 |  |  | 116.3 |  | 89.4 |  | 34.4 |  | 63.5 |
| 60 |  |  | | 180 |  |  | 129.7 |  | 104.5 |  | 53.7 |  | 66.2 |
| 120 |  |  | | 360 |  |  | 132.8 |  | 103.5 |  | 54.6 |  | 83.9 |
| 180 |  |  | | 540 |  |  | 144.2 |  | 126.6 |  | 76.0 |  | 92.0 |
| P-value | | | | | |  | ≤ 0.10 |  | ≤ 0.01 |  | ≤ 0.01 |  | ≤ 0.001 |
| Regression | | | | | |  | y=117.7+0.14x  R²: 0.95% |  | y=89.4+0.18x  R²: 86% |  | y=35.8+0.21x  R²: 91% |  | y=60.9+0.17x  R²: 93% |
| **N accumulation in stalks (kg ha^-1^)** | | | | | | | | | | | | | |
| 0 |  |  | | 0 |  |  | 100.5 |  | 43.4 |  | 51.6 |  | 41.8 |
| 60 |  |  | | 180 |  |  | 103.0 |  | 58.1 |  | 65.0 |  | 51.6 |
| 120 |  |  | | 360 |  |  | 98.5 |  | 59.4 |  | 51.3 |  | 60.4 |
| 180 |  |  | | 540 |  |  | 97.4 |  | 109.8 |  | 55.1 |  | 70.0 |
| P-value | | | | | |  | 0.91 |  | ≤ 0.001 |  | 0.46 |  | ≤ 0.0001 |
| Regression | | | | | |  | - |  | y=37.6+0.33x  R²: 79% |  | - |  | y=41.9+0.15x  R²: 99% |
| **N accumulation in tops (kg ha^-1^)** | | | | | | | | | | | | | |
| 0 |  |  | | 0 |  |  | 43.9 |  | 40.4 |  | 30.9 |  | 58.3 |
| 60 |  |  | | 180 |  |  | 42.4 |  | 32.8 |  | 42.4 |  | 79.5 |
| 120 |  |  | | 360 |  |  | 39.5 |  | 42.0 |  | 40.1 |  | 81.0 |
| 180 |  |  | | 540 |  |  | 39.1 |  | 38.2 |  | 31.6 |  | 128.0 |
| P-value | | | | | |  | 0.79 |  | 0.70 |  | 0.33 |  | ≤ 0.0001 |
| Regression | | | | | |  | - |  | - |  | - |  | y=55.1+0.35x  R²: 85% |
| **N accumulation in dry leaves (kg ha^-1^)** | | | | | | | | | | | | | |
| 0 |  |  | | 0 |  |  | 15.7 |  | 10.4 |  | 6.5 |  | 15.7 |
| 60 |  |  | | 180 |  |  | 15.8 |  | 9.7 |  | 8.5 |  | 15.0 |
| 120 |  |  | | 360 |  |  | 18.8 |  | 11.4 |  | 6.3 |  | 12.9 |
| 180 |  |  | | 540 |  |  | 25.8 |  | 13.1 |  | 6.3 |  | 18.1 |
| P-value | | | | | |  | ≤ 0.001 |  | 0.52 |  | ≤ 0.01 |  | 0.22 |
| Regression | | | | | |  | y=14.03+0.05x  R²: 82% |  | - |  | y=-0.0001x^2^+0.02x+6.82;  R²: 40% | | - |
| **N accumulation in the whole plant (kg ha^-1^)** | | | | | | | | | | | | | |
| 0 |  |  | | 0 |  |  | 160.1 |  | 94.2 |  | 89.1 |  | 115.8 |
| 60 |  |  | | 180 |  |  | 161.2 |  | 100.6 |  | 116.0 |  | 146.1 |
| 120 |  |  | | 360 |  |  | 156.8 |  | 112.8 |  | 97.7 |  | 154.3 |
| 180 |  |  | | 540 |  |  | 162.2 |  | 161.1 |  | 93.0 |  | 216.1 |
| P-value | | | | | |  | 0.94 |  | 0.06 |  | 0.24 |  | ≤ 0.0001 |
| Regression | | | | | |  | - |  | - |  | - |  | y=111.71+0.51x  R²:89% |

^1^Means were compared by the Regression-Test (P < 0.05). When significant, regression models (linear or quadratic) are indicated.

**Tab. 3S** N accumulation, N derived from fertilizer (NDF), and N derived from soil (NDS) in each plant compartment in the 4^th^ ratoon, in the microplots that received 100 kg ha^-1^ of ^15^N^1^.

| **N rates, kg ha^-1^** | | | | | |  | **Plant compartments** | | | | | | | | | | |
| --- | --- | --- | --- | --- | --- | --- | --- | --- | --- | --- | --- | --- | --- | --- | --- | --- | --- |
| **1^st^ to 3^rd^** | | **4^th^** | | **Total** | |  | **Dry leaves** | | | **Tops** | | | **Stalks** | | | **Total** | |
| **N accumulation (****kg ha^-1^)** | | | | | | | | | | | | | | | | | |
| 0 |  | 100 |  | 100 |  |  | 16.6 | b | 109.3 | | c | 76.0 | |  | 201.9 | |  |
| 180 |  | 100 |  | 280 |  |  | 16.0 | b | 132.8 | | a | 64.5 | |  | 213.3 | |  |
| 360 |  | 100 |  | 460 |  |  | 21.4 | a | 126.1 | | ab | 65.2 | |  | 212.7 | |  |
| 540 |  | 100 |  | 640 |  |  | 22.0 | a | 117.3 | | bc | 74.2 | |  | 213.5 | |  |
| P-value | |  |  |  |  |  | <0.01 |  | <0.002 | |  | 0.09 | |  | 0.26 | |  |
| **NDF (kg ha^-1^)** | | | | | | | | | | | | | | | | | |
| 0 |  | 100 |  | 100 |  |  | 3.1 | b | 21.3 | | a | 16.3 | | a | 40.7 | | a |
| 180 |  | 100 |  | 280 |  |  | 2.6 | b | 19.9 | | ab | 9.6 | | b | 32.1 | | b |
| 360 |  | 100 |  | 460 |  |  | 2.9 | b | 18.0 | | bc | 9.6 | | b | 30.5 | | b |
| 540 |  | 100 |  | 640 |  |  | 4.6 | a | 16.4 | | c | 10.6 | | b | 31.6 | | b |
| P-value | |  |  |  |  |  | <0.01 |  | <0.01 | |  | <0.001 | |  | <0.001 | |  |
| **NDS (kg ha^-1^)** | | | | | | | | | | | | | | | | | |
| 0 |  | 100 |  | 100 |  |  | 13.5 | b | 88.0 | | c | 59.7 | |  | 161.2 | | b |
| 180 |  | 100 |  | 280 |  |  | 13.4 | b | 113.0 | | a | 54.8 | |  | 181.2 | | a |
| 360 |  | 100 |  | 460 |  |  | 18.6 | a | 108.1 | | ab | 55.5 | |  | 182.2 | | a |
| 540 |  | 100 |  | 640 |  |  | 17.4 | a | 100.9 | | b | 63.6 | |  | 181.9 | | a |
| P-value | |  |  |  |  |  | ≤ 0.01 |  | ≤ 0.01 | |  | 0.33 | |  | ≤ 0.01 | |  |

^1^Averafes followed by different letters in the column differ statistically according to the LSD-Test (P ≤ 0.05).


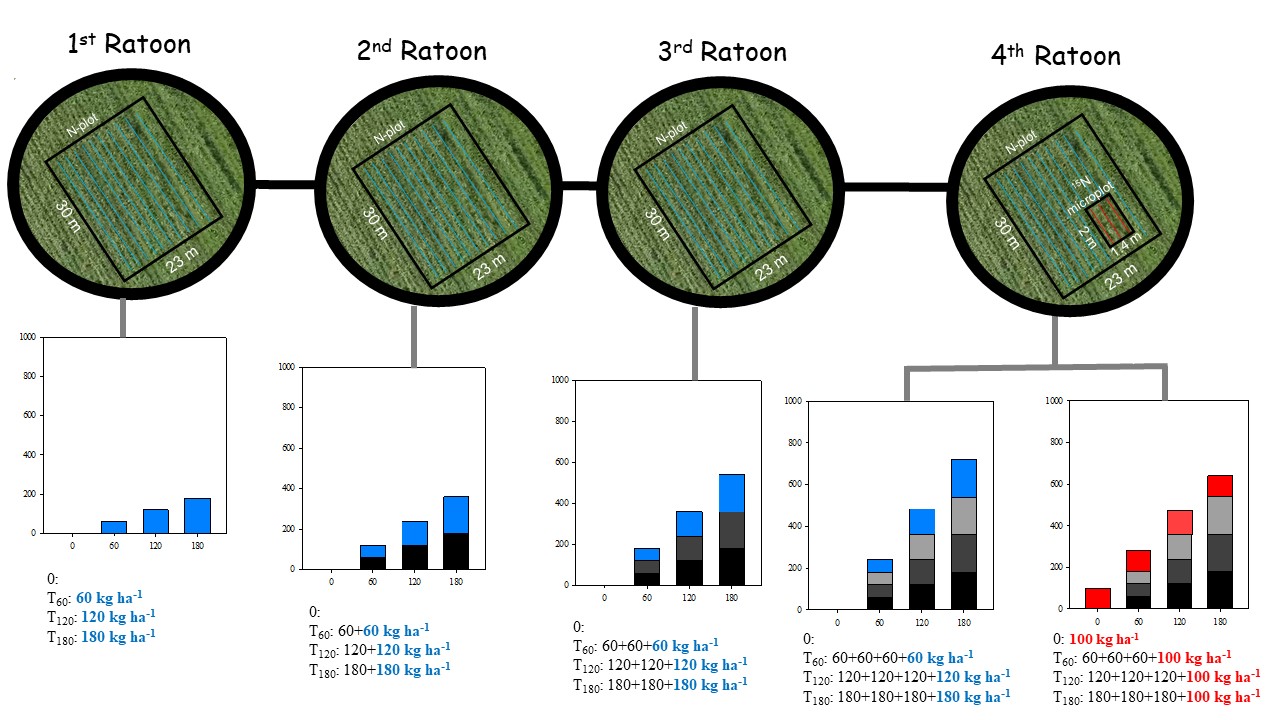


**Fig. 1S** Details of annual application of N rates (60, 120, 180 kg ha^-1^) to sugarcane ratoons. In the 4^th^ ratoon, the plots were split; half received the same N rates of the previous years, and half were fertilized with 100 kg ha^-1^ (Bars in red). Microplots of ^15^N-labeled fertilizer were set up within the plots receiving 100 kg ha^-1^ N. X and Y axes refer to annual N rates and cumulative N, respectively.
